# Structural and Functional Analyses of the Transcription Repressor DgoR From *Escherichia coli* Reveal a Divalent Metal-Containing D-Galactonate Binding Pocket

**DOI:** 10.3389/fmicb.2020.590330

**Published:** 2020-11-05

**Authors:** Zhaozhu Lin, Yi Sun, Yu Liu, Shujuan Tong, Zhuo Shang, Yuanheng Cai, Wei Lin

**Affiliations:** ^1^Department of Microbiology and Immunology, School of Medicine & Holistic Integrative Medicine, Nanjing University of Chinese Medicine, Nanjing, China; ^3^Biochemistry and Cell Biology Department, Stony Brook University, Stony Brook, NY, United States; ^2^Department of Chemistry, Waksman Institute of Microbiology, Rutgers University, Piscataway, NJ, United States; ^4^State Key Laboratory of Natural Medicines, China Pharmaceutical University, Nanjing, China; ^5^Jiangsu Collaborative Innovation Center of Chinese Medicinal Resources Industrialization, Nanjing, China

**Keywords:** DgoR, metal binding site, D-galactonate, FadR family, *Escherichia coli*, transcription repressor

## Abstract

The transcription repressor of D-galactonate metabolism, DgoR, from *Escherichia coli* belongs to the FadR family of the GntR superfamily. In the presence of D-galactonate, DgoR binds to two inverted repeats overlapping the *dgo* cis-acting promoter repressing the expression of genes involved in D-galactonate metabolism. To further understand the structural and molecular details of ligand and effector interactions between D-galactonate and this FadR family member, herein we solved the crystal structure of C-terminal domain of DgoR (DgoR_C), which revealed a unique divalent metal-containing substrate binding pocket. The metal ion is required for D-galactonate binding, as evidenced by the dramatically decreased affinity between D-galactonate and DgoR in the presence of EDTA, which can be reverted by the addition of Zn^2+^, Mg^2+^, and Ca^2+^. The key amino acid residues involved in the interactions between D-galactonate and DgoR were revealed by molecular docking studies and further validated with biochemical studies by site-directed mutagenesis. It was found that changes to alanine in residues R102, W181, T191, and R224 resulted in significantly decreased binding affinities for D-galactonate, as determined by EMSA and MST assays. These results suggest that the molecular modifications induced by a D-galactonate and a metal binding in the DgoR are required for DNA binding activity and consequently, transcriptional inhibition.

## Introduction

Bacteria have evolved to swiftly adapt to physical and chemical changes in environment through fine-tuning of their metabolic processes. Such processes are predominantly controlled at the transcription level, by which gene transcription rates of the operon that encodes key enzymes involving in appropriate metabolic pathways are exquisitely regulated. Those operons are typically regulated by transcription factors that bind substrates or products of the pathways (Balleza et al., 2008; Belliveau et al., 2018).

One of the most widespread transcription factors that regulate transcription through binding to metabolites is the GntR superfamily, which was first described in 1991 and named by the gluconate operon repressor in *Bacillus subtilis* (Haydon and Guest, 1991). Most members of this superfamily are known to regulate many fundamental cellular processes, such as motility (Jaques and McCarter, 2006), development (Hoskisson et al., 2006), antibiotic production (Ostash et al., 2011), antibiotic resistance (Jaques and McCarter, 2006), plasmid transfer (Reuther et al., 2006), and virulence (Casali et al., 2006). GntR superfamily transcription factors exert their functions through allosteric regulation, where the binding of the effector molecule alters the binding affinity of the transcription factor to its operon. Members of GntR superfamily share a similar winged-helix-turn-helix (wHTH) DNA-binding domain at the N-terminus (NTD), but different effector-binding and oligomerization domains at the C-terminus (CTD). The diversity of the CTD domain can subdivide the GntR superfamily into six major families: HutC, MocR, YtrA, AraR, PlmA, and FadR (Rigali et al., 2002; Lee et al., 2003; Zheng et al., 2009; Jain, 2015). As the representative member of GntR superfamily, FadR family is characterized by the presence of a helical domain at the C-terminus (FCD domain). Most members of FadR family are involved in the transcriptional regulation of enzymes responsible for substrate oxidation in amino acid metabolism (Haydon and Guest, 1991; van Aalten et al., 2000, 2001b; Campbell and Cronan, 2001; Xu et al., 2001; Rigali et al., 2002). Although the crystal structures of several members of the GntR superfamily are well-described and publicly available, the information on their sensory ligands and ligand binding pockets is very scarce so far.

Bacteria can utilize a variety of sugar acids as carbon sources to adapt to different physicochemical conditions in the environment. The metabolism of D-galactonate, a widely prevalent aldonic sugar acid, is considered to be important for the enteric bacterium *E. coli*. It metabolizes D-galactonate through the enzymes encoded by D-galactonate operon (*dgo*) and a modified Entner-Doudoroff pathway (Peekhaus and Conway, 1998), where D-galactonate is degraded into D-glyceraldehyde-3-phosphate and pyruvate, and then enter central metabolism (Singh et al., 2019; Arya et al., 2020). DgoR is encoded by the first gene of *dgo* operon and negatively regulates the whole operon. DgoR was previously predicted to be a FadR family of transcription regulator, and repress the D-galactonate metabolic pathway by binding inverted repeats in the *dgo* cis-acting element using D-galactonate as a specific effector molecule (De Ley and Doudoroff, 1957; Deacon and Cooper, 1977; Cooper, 1978; Singh et al., 2019; Arya et al., 2020). Although several regulators of the FadR family have been well described at the molecular level, the binding pocket of D-galactonate and its substrate specificity have not been investigated (Singh et al., 2019).

In our study, we systematically investigated the D-galactonate binding pocket of DgoR using X-ray crystallography, Electrophoretic Mobility Shift Assay (EMSA), and Micro Scale Thermophoresis (MST) assays. Meanwhile, a molecular docking model of the C-terminal domain of *E. coli* DgoR (*Eco*DgoR_C) complexed with D-galactonate was established based on the crystal structure of *Eco*DgoR_C. The key amino acid residues in the binding pocket that are involved in the interactions with D-galactonate were predicted and further validated by site-directed mutational analysis. Moreover, our study shows that some divalent cations (e.g., Zn^2+^, Mg^2+^ and Ca^2+^) are essential for the interaction between DgoR and D-galactonate, but also reveals a unique divalent metal-containing substrate binding pocket, which is the largest one in the GntR superfamily reported so far. The structural information on substrate-enzyme interactions derived from our experimental and docking studies provides a better understanding of the structural and molecular details of effector interactions between sugar acid and the FadR family member.

## Materials and Methods

### Gene Cloning, Site-Directed Mutagenesis, Expression, and Protein Purification

Wild-type *dgoR* gene and *dgoR_C* from *Escherichia coli* were cloned into the pET28a vector under control of the bacteriophage T7 gene promoters using *Nhe*I and *Hind*III. The resulting plasmids were transformed into *E. coli* strain BL21 (DE3) (Invitrogen). Single colonies of the resulting transformants were used to inoculate 50 mL of LB broth containing 50 μg/mL kanamycin, followed by incubating at 37°C, 180 rpm for 16 h. Aliquots (10 mL) were used to inoculate 1 L of LB broth containing 50 μg/mL kanamycin. The cultures were shaken at 37°C, 180 rpm, and 1 mM isopropyl-β-D-thiogalactoside was added into the culture when OD_600_ reached to 0.8. After incubation at 16°C, 180 rpm for 16 h, cells were harvested by centrifugation (4,000 × g; 15 min at 4°C), re-suspended in buffer A (10 mM Tris-HCl, pH 8.0, 200 mM NaCl, 5 mM DTT, and 5% glycerol), and lysed using an EmulsiFlex-C5 cell disruptor (Avestin). The lysate was centrifuged (20,000 × g; 30 min at 4°C) and the cell debris were discarded. The supernatant was loaded onto a 5 mL column of Ni^2+^-NTA-agarose (Qiagen) pre-equilibrated with buffer A, and the column was washed with 10 × 5 ml buffer A containing 25 mM imidazole followed by eluting with 50 mL buffer A containing 250 mM imidazole. The eluted raw protein was concentrated to around 10.0 mg/mL and further purified by gel filtration chromatography on a HiLoad 16/60 Superdex 200 prep grade column (GE Healthcare) by the solution comprising of 20 mM Tris-HCl (pH 8.0), 100 mM NaCl, 5 mM MgCl_2_, and 1 mM β-mercaptoethanol. The target peak was collected and concentrated to 10 mg/mL in the same buffer using 10 kDa MWCO Amicon Ultra-15 centrifugal ultrafilters (EMD Millipore), and stored in aliquots at -80°C. Yields were ∼5 mg/L, and purities were ∼95%. Site-directed mutagenesis of *dgoR* or *dgoR_C* were prepared using a one-step PCR method. The mutated proteins were expressed and purified using the same method as described above. Supplementary Table S1 provides a list of bacterial strains, plasmids, and primers in the study.

### Preparation of D-Galactonate

D-galactonate was prepared from its calcium salt as described previously (Singh et al., 2019). Briefly, equivalent amounts of calcium D-galactonate and oxalic acid were mixed in boiling water and vortexed for 3 min. The milky solution was filtered through a 0.2 μm filter. The filtrate was immediately transferred to 4°C for 15 min. Crystals were collected and dried overnight at room temperature on a Whatman filter paper. Crystals of D-galactonate were stored at room temperature.

### Electrophoretic Mobility Shift Assay (EMSA)

For expression of the recombinant DgoR mutants (i.e., R102A, D146A, H150A, Q173A, R179A, W181A, D184A, T191A, H195A, S221A, R224A, and R224E), the wild-type *E. coli dgoR* gene on the expression plasmid pET28a was mutated using the site-directed mutagenesis method. The *dgo* cis-acting element region of *E. coli* K12 was generated by PCR and subsequently inserted into the *Hinc*II site of pUC18. The obtained plasmid was used as the template for preparation of the Cy5-labeled probes using the universal primer pair of Cy5-labeled *dgoR*cis-acting_F and *dgoR*cis-acting_R. Cy5-labeled probe (100 ng) was incubated with different amounts of *dgoR* or its mutants at 25°C for 20 min in a buffer comprising of 25 mM Tris-HCl (pH 8.0), 50 mM KCl, 2.5 mM MgCl_2_, 5% glycerol, 1 mM dithiothreitol (DTT), and 100 μg/mL sonicated salmon sperm DNA (Sangon) (total volume 20 μl). The resulting DNA-protein complexes were subjected to electrophoresis on native PAGE gels at 100 V, 4°C for 2.5 h in a running buffer containing 25 mM Tris-HCl (pH 7.8) and 250 mM glycine. After electrophoresis, gels were directly scanned for fluorescent DNAs using an ImageQuantTM LAS 4000 system (GE Healthcare). For EMSAs in the presence or absence of D-galactonate or D-glucose, 0.5 mM D-galactonate or D-glucose was applied.

### Microscale Thermophoresis (MST) Binding Assays

His-tagged DgoR and its derivatives were labeled with the NT-647-NHS dye using the Monolith NT^TM^ Protein Labeling Kit RED-NHS (NanoTemper Technologies) (Magnez et al., 2017; Welsch et al., 2017). Serially diluted unlabeled proteins and 100 nM of labeled DgoR or its derivatives were incubated for 15 min at room temperature in binding buffer (1.8 mM KH_2_PO_4_, 10 mM Na_2_HPO_4_, 137 mM NaCl, 2.7 mM KCl, and 0.05% Tween-20, pH 7.8) in a final volume of 20 μL. Subsequently, samples were loaded into NT.115 premium coated capillaries (NanoTemper Technologies). Binding experiments were performed using a Monolith NT.115 Pico apparatus (NanoTemper Technologies) with the following parameters: LED power 5 %, MST Power high. MST traces were analyzed between 4.00 and 5.00 s after turning on the IR-Laser. Measurements were carried out with D-galactonate. For each experiment, the unlabeled proteins were produced from independent preparations. Results were obtained with the MO Control software version 1.6. MO Affinity Analysis software version 2.3 was used to determine the fraction of the formed complex. Apparent dissociation constants (K_*d*_) were calculated using nonlinear fitting assuming one specific binding site with the GraphPad Prism 7 software with the following formula

Y=BXMax*/KD+X

(whereBisMaxthemaximumtheoreticalspecificbinding,hereB=Max1).

### Crystallization of *Eco*DgoR_C

Robotic crystallization trials were performed for *Eco*DgoR_C using a Gryphon liquid handling system (Art Robbins Instruments), commercial screening solutions (Emerald Biosystems, Hampton Research, and Qiagen), and the sitting-drop vapor-diffusion technique (drop: 0.2 μL *Eco*DgoR_C plus 0.2 μL screening solution; reservoir: 60 μL screening solution; 22°C). 900 conditions were screened in total. Under several conditions, *Eco*DgoR_C crystals appeared within 1 week. Conditions were optimized using the hanging-drop vapor-diffusion technique at 22°C. The optimized crystallization condition for DgoR_C was as follows: 0.1 M sodium acetate/acetate acid (pH 5.5), 2 M lithium sulfate, 0.1 M magnesium sulfate, 5% (*v*/*v*) PEG 400 at 22°C; crystals were transferred into the reservoir solution containing 18% (*v*/*v*) (2*R*,3*R*) -(-)-2,3-butanediol (Sigma-Aldrich) and flash-cooled with liquid nitrogen.

### Structure Determination and Refinement of *Eco*DgoR_C

X-ray diffraction data of *Eco*DgoR_C were collected from cryo-cooled crystals at SSRF Beamline BL17U1 (Shanghai Synchrotron Radiation Facility). Data were processed using HKL2000 and CCP4i programs (Collaborative Computational Project, Number 4, 1994; Potterton et al., 2018). The resolution cut-off criteria were as below: (i) I/σ ≥ 1.0, (ii) CC_1/2_ (highest resolution shell) >0.5. The AutoSol program of Phenix was used to search the anomalous signals of selenium atoms and to calculate the phase and the initial model of the *Eco*DgoR_C was built using the Autobuild implemented in Phenix (Adams et al., 2010). Over 60% of main chain residues were built, and the overall figure of merit was increased from 0.325 to 0.428. The initial model was then used as a guide to build the remainder of the protein manually into density-modified electron density maps with the program Coot (Emsley and Cowtan, 2004). The full structure model was completed by iterative manual building in Coot (Emsley and Cowtan, 2004) and refined with Phenix and Refmac. Water molecules were automatically and/or manually added to the model. The final model of *Eco*DgoR_C was refined to 2.0 Å resolution with R_*work*_ and R_*free*_ values of 0.22 and 0.24 (Table 1), and deposited in PDB with an accession number of **7C7E**.

**TABLE 1:** Structure data collection and refinement statistics.

Protein	*Eco*DgoR_C
PDB code	7C7E
Data collection source	SSRF BL17U
**Data collection**	
Wavelength	0.97917
Space group	P6_5_22
Cell dimensions	
a, b, c (Å)	85.037,85.037,111.688
α, β, γ (°)	90.0, 90.0, 120.0
Resolution (Å)	73.65–2.05 (2.10–2.05)*
Number of unique reflections	15,623
R_*merge*_	0.091 (1.243)*
R_*meas*_	0.093 (1.278)*
R_*pim*_	0.015 (0.265)*
CC_1/2_ (highest resolution shell)	0.783
I/σI	34.6 (2.40)*
Completeness (%)	100.0 (100.0)*
Multiplicity	33.2 (18.4)
Anomalous completeness	100.0 (100.0)
Anomalous multiplicity	18.1 (9.6)
**Refinement**	
Number of unique reflections	27,293
Number of test reflections	1,406
R_*work*_/R_*free*_	0.22/0.24 (0.29/0.33)*
Number of atoms	
Protein	1,165
Water	35
r.m.s. deviations	
Bond lengths (Å)	0.003
Bond angles (°)	0.553
MolProbity statistics	
Clashscore	2.71
Rotamer outliers (%)	0
Cβ outliers (%)	0
Ramachandran plot	
Favored (%)	95
Outliers (%)	0

*Highest resolution shell in parentheses.

### Molecular Docking Study

All molecular docking studies were performed using Autodock 4.2 package (Morris et al., 2009). Briefly, chain A of *Eco*DgoR_C crystal structure was used as the rigid molecule. The molecule was added with non-polar hydrogens and assigned partial atomic charges using AutoDockTools (ADT) (Morris et al., 2009). The coordinates of D-galactonate were generated using CORINA Classic online service. A grid box with 80 × 80 × 100 grid points and 0.2 Å grid spacing centered roughly at the DgoR substrate binding pocket was used as the searching space. 100 runs of Larmarckian Genetic Algorithm were performed to search the protein-ligand interactions. The results were clustered and ranked. Result analyses and figure rendering were performed using PyMOL.

### Data Availability

The structure of *Eco*DgoR_C has been deposited into Protein Data Bank (PDB) with the accession number of **7C7E**.

## Results

### Crystal Structure of *Eco*DgoR_C

An early study reported that D-galactonate, an aldonic sugar acid, is the inducer of the *dgo* operon that is responsible for D-galactonate metabolism in *E. coli* through direct binding to *Eco*DgoR (Gao et al., 2008; Zheng et al., 2009). Recently, the molecular and functional basis for the regulation of D-galactonate metabolism in *Eco*DgoR have been revealed (Singh et al., 2019). To confirm these results in our EMSA system, we incubated *Eco*DgoR with its *dgo* cis-acting element in the presence of 0.5 mM D-galactonate and tested its electrophoretic mobility by EMSA experiments. Moreover, D-glucose was examined as a negative control in order to confirm the specificity of D-galactonate to *Eco*DgoR. Our results showed that D-galactonate relieved DNA bound by *Eco*DgoR in a concentration-dependent manner, while D-glucose did not display such effect (Supplementary Figure S1), which is highly consistent with previous conclusion that D-galactonate is a specific effector of *Eco*DgoR (Gao et al., 2008; Zheng et al., 2009; Singh et al., 2019).

**FIGURE 1:**
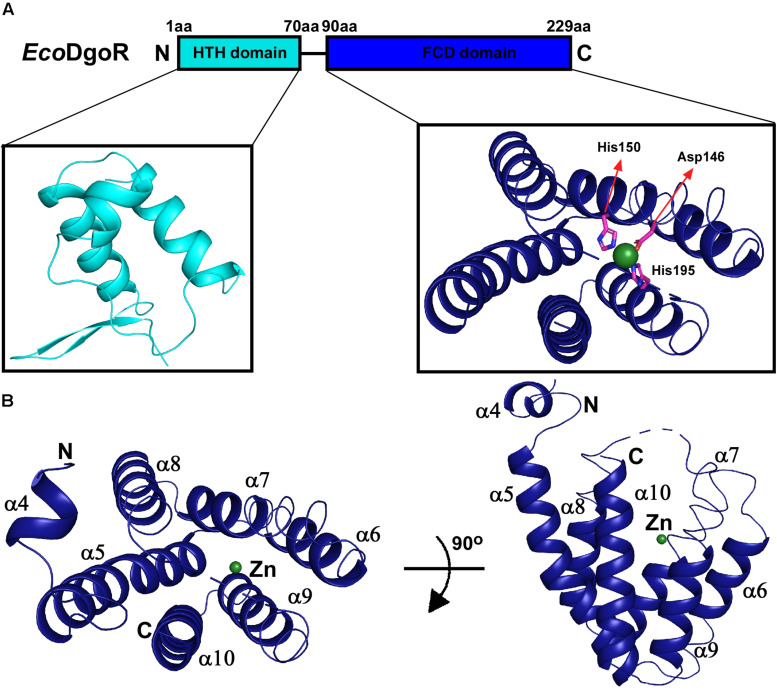
Domain organization and structure of the wide-type *Eco*DgoR. **(A)** Structural organization of *Eco*DgoR. N-terminal helix turn helix DNA binding domain of *Eco*DgoR (*Eco*DgoR_N) and C-terminal FCD domain of *Eco*DgoR (*Eco*DgoR_C) are in cyan and blue, respectively. The structure of N-terminal DNA binding domain was modeled based on the structure of *Tm*0439 (PDB: 3fms) from *Thermotoga maritima*, and the structure of *Eco*DgoR_C was solved in this study (PDB: 7C7E). A green sphere indicates the divalent metal ion found in the structure of *Eco*DgoR_C. **(B)** Overall structure of *Eco*DgoR_C. *Eco*DgoR_C is colored in blue and two orthogonal views are shown.

**FIGURE 2:**
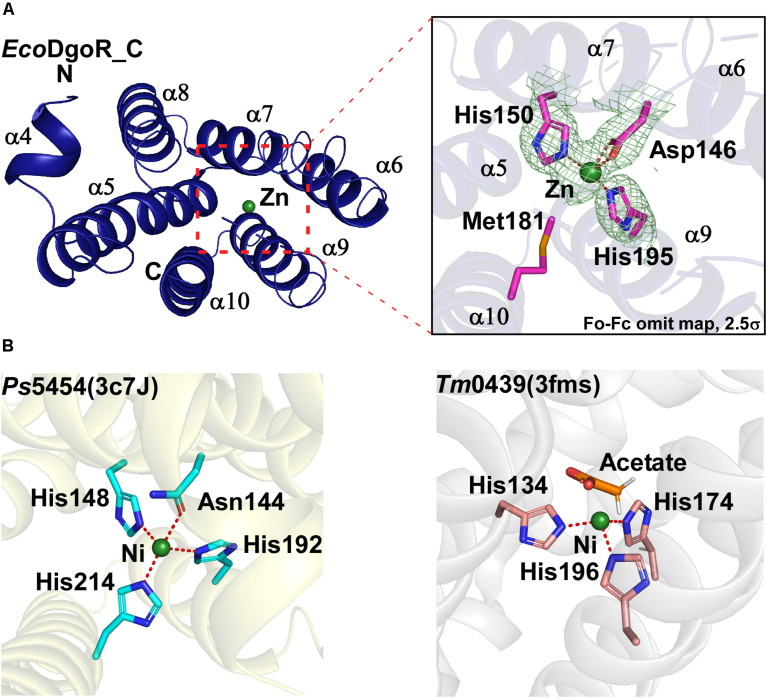
Metal ion binding site of the wide-type *Eco*DgoR and its GntR family homologues. **(A)** Close-up view of the metal binding site of *Eco*DgoR. The amino acid residues involved in Zn^2+^ coordination are shown as purple/blue sticks, and Zn^2+^ as a green sphere; **(B)** Left panel, close-up view of the metal binding site of *Ps*5454 (PDB: 3c7J), GntR superfamily protein from *Pseudomonas syringae pv. syringae*. The amino acid residues involved in Ni^2+^ binding are shown as cyan/blue sticks, and Ni^2+^ as a green sphere; right panel, close-up view of the metal binding site of *Tm*0439 (PDB: 3fms), GntR superfamily protein from *Thermotoga maritima* strain ATCC 43589. The amino acid residues coordinating Ni^2+^ are shown as pink/blue sticks, and Ni^2+^ as a green sphere.

*Eco*DgoR consists of two domains, an N-terminal winged helix-turn-helix domain (*Eco*DgoR_N) and a C-terminal domain of *Eco*DgoR (*Eco*DgoR_C) based on the sequence alignment results (Figure 1A and Supplementary Figure S2). The amino acid sequence similarity between *Eco*DgoR and other reported FadR members *Tm*0439, *Cgl*LIDR, and *Ps*5454 are 20.99, 23.85, and 32.88%, respectively (Supplementary Figure S2) (Gao et al., 2008; Zheng et al., 2009). Our attempt to obtain crystal structure of the complete *Eco*DgoR was unsuccessful. Thus, we set out to crystalize the C-terminal domain of *Eco*DgoR (*Eco*DgoR_C) and successfully obtained its crystal structure at 2.2 Å resolution. Statistics of data collection and model refinement are summarized in Table 1. The *Eco*DgoR_C, encompassing residues 90–229, contains seven α-helices which arranged into an antiparallel bundle (Figure 1B). An internal polar cavity in *Eco*DgoR_C was proposed to be D-galactonate binding pocket, and the bottom of which includes three residues Asp146, His150 and His195 with their side chains arranged in a three-blade propeller shape and the nitrogen and oxygen atoms pointing toward a strong peak of positive electron density (Figure 2A). When a dummy atom (i.e., Zn^2+^, Ni^2+^) was placed in this density and refined, it was found to be 2.0–2.2 Å distance from the three surrounding residues, which is consistent with the coordination stereochemistry of a divalent metal ion.

### Molecular Docking Model of D-Galactonate-Divalent Metal Ion-*Eco*DgoR Complex

A plethora of biochemical evidence suggested that the majority of C-terminal domains of FadR family transcription regulators are metal (most likely Zn^2+^) dependent (van Aalten et al., 2000, 2001a, b; Campbell and Cronan, 2001; Xu et al., 2001; Rigali et al., 2002; Gao et al., 2008; Blancato et al., 2016). It has been reported that metal-sensing transcription factors are ubiquitous in prokaryotes, with seven major families (i.e., ArsR, MerR, CopY, Fur, DtxR, CsoR, and NikR) characterized to date (Gao et al., 2008; Zheng et al., 2009). Almost all of these proteins are dimeric and typically bind metal ions at or near their dimer interfaces. The metal ions modulate the regulator proteins to repress, de-repress, or activate the transcription of operons coding for metal-efflux pumps, transporters, redox machinery, and so on (Rigali et al., 2002). In contrast, the metal ion-binding sites of FadR family proteins are buried within an individual protomer, and the removal of metal ions from FadR family proteins is relatively more difficult than those from metal-sensing regulator proteins (Gao et al., 2008; Zheng et al., 2009). These significant differences inferred that metal ions may play structural roles in effector binding/coordination of FadR family proteins.

In the crystal structure of *Eco*DgoR_C, two imidazole groups of His150 and His195 along with Asp146 form a three-blade propeller shape, which highly likely coordinates Zn^2+^ suggested from the refined B value (36 Å^2^) (Figure 2A). Similarly, two FadR family proteins *Ps*5454 and *Tm*0439 coordinate Ni^2+^ in stereochemically analogous sites. In *Ps*5454, three imidazole groups of His148, His192, and His214, along with Asn144 (equivalent to Asp146 in *Eco*DgoR), are involved in Ni^2+^ binding. While in *Tm*0439, His134, His174, and His196, as well as the ligand acetate may play the role as the fourth residue to coordinate Ni^2+^ (Figure 2B). Surprisingly, FadR, the typical FadR family protein, does not contain any metal ion-binding sites, because the corresponding sites of amino acid residues for metal ion coordination (i.e., His) are replaced by Phe149, Tyr193, and Tyr215, none of which is suitable for metal ion binding (Supplementary Figure S2).

**FIGURE 3:**
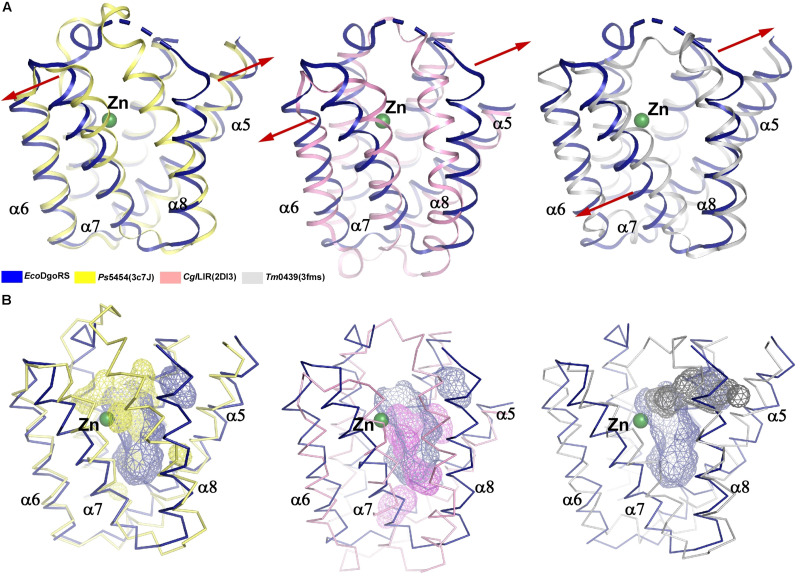
Comparison of substrate binding pockets of the wide-type *Eco*DgoR_C, *Ps*5454_C, *Cgl*LIDR_C, and *Tm*0439_C. **(A)** Structure superimposition of *Eco*DgoR_C with *Ps*5454_C (left), *Cgl*LIDR_C (middle), and *Tm*0439_C (right). Structures of *Eco*DgoR_C, *Ps*5454_C, *Cgl*LIDR_C and *Tm*0439_C are shown with cartoons colored in blue, yellow, pink, and gray, respectively. **(B)** Comparison of substrate binding pockets of *Eco*DgoR_C with *Ps*5454_C (left), *Cgl*LIDR_C (middle), and *Tm*0439_C (right). Structures of *Eco*DgoR_C, *Ps*5454_C, *Cgl*LIDR_C, and *Tm*0439_C are shown with ribbons colored in blue, yellow, pink and gray, respectively, Substrate binding pockets of *Eco*DgoR_C, *Ps*5454_C, *Cgl*LIDR_C, and *Tm*0439_C are shown with surface colored in blue, yellow, pink and gray, respectively. The surfaces were generated in Pymol and the volumes of pockets were calculated using CASTp analysis (Tian et al., 2018).

**FIGURE 4:**
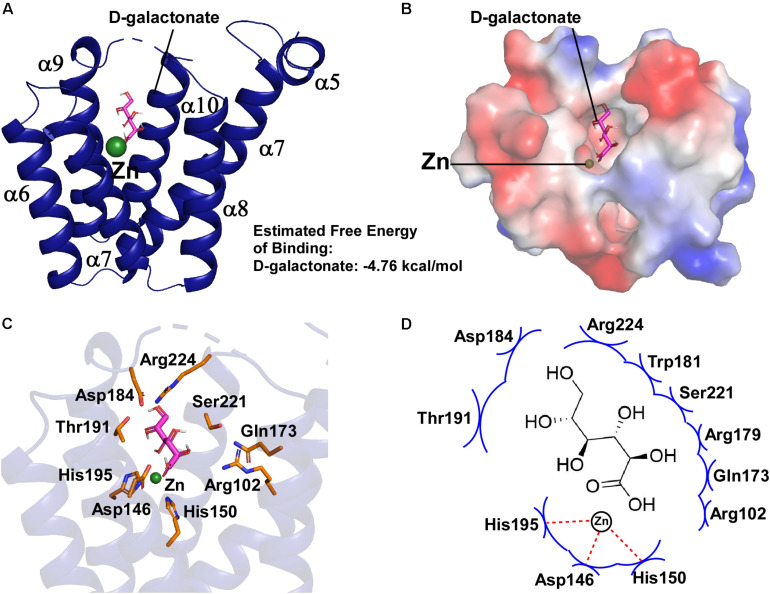
Molecular docking of D-galactonate to *Eco*DgoR_C. **(A)** Energetically favorable docking model of D-galactonate to *Eco*DgoR_C. D-Galactonate is shown as stick models colored in magenta; green sphere, metal atom. **(B)** Surface electrostatic potential of *Eco*DgoR_C in complex with D-galactonate. **(C)** Close-up views of simulated D-galactonate-*Eco*DgoR_C interactions in the binding pocket. Interactions residues are shown as stick models colored in orange. **(D)** Summary of the predicted D-galactonate-*Eco*DgoR_C interaction where the key amino acid residues involving in the interactions are labeled. Red dashed line, hydrogen bond; blue arcs, van der Waals interaction.

By analyzing the crystal structure of *Eco*DgoR_C, we found that the buried solvent-accessible volumes of *Eco*DgoR_C (∼1,532 Å^3^) is significantly larger than that of *Ps*5454, *Cgl*LIDR, and *Tm*0439 (∼980, 1,096, and 756 Å^3^, respectively) (Figure 3). It is, therefore more plausible that the *Eco*DgoR_C binds carboxylic acids (e.g., D-galactonate) so that the latter are buried in the ligand-binding cavity and interact directly with the metal ions. To define the potential D-galactonate binding pocket of *Eco*DgoR and uncover the possible role of divalent metal ion in the ligand-transcription factor interaction, we attempted to co-crystallize *Eco*DgoR_C with D-galactonate and soak D-galactonate into the apo *Eco*DgoR_C crystals. However, our trials were unsuccessful probably due to crystallizing or improper crystal packing. Alternatively, *in silico* molecular docking approach was used to simulate the interaction between *Eco*DgoR_C, D-galactonate as well as the metal ion. The docking results showed that D-galactonate can occupy the polar cavity at the bottom of *Eco*DgoR_C with an estimated free binding energy of -4.76 kcal/mol. In the molecular docking model, D-galactonate is surrounded by a hydrophobic residue W181, and hydrophilic residues R102, D146, H150, Q173, R179, D184, T191, H195, S221, and R224 (Figure 4). Furthermore, the residues Asp184, Thr191, Ser221, and Arg224 in the binding pocket may interact with D-galactonate through hydrogen bonds (Figure 4). Overall, the molecular docking model of D-galactonate-divalent metal ion-*Eco*DgoR complex reveals that the relatively large polar cavity located in the center of *Eco*DgoR_C could accommodate the bulky sugar acid molecule, D-galactonate, which may interact with the divalent metal ion (e.g., Zn^2+^) directly.

### Key Amino Acid Residues of D-Galactonate Binding Pocket in *Eco*DgoR, and the Divalent Metal Cation-Mediated Interactions of *Eco*DgoR and D-Galactonate

We next sought to experimentally evaluate and verify the interactions between D-galactonate and *Eco*DgoR predicted from the molecular docking study. Eleven residues within 5 Å to the D-galactonate molecule (R102, D146, H150, Q173, R179, W181, D184, T191, H195, S221, and R224) were identified to potentially interact with D-galactonate. Particularly, seven residues (D146, H150, H195, R102, Q173, R179, and T191) were located in the proximity of both D-galactonate molecule and Zn^2+^. Based on these observations, we hypothesized that the divalent cation may facilitate ligand-protein binding or shape the conformation of binding pocket. Thus, we generated 12 single mutations at these sites, and assessed the effects of mutations on the binding affinities of *Eco*DgoR to its cis-acting elements by EMSA.

**FIGURE 5:**
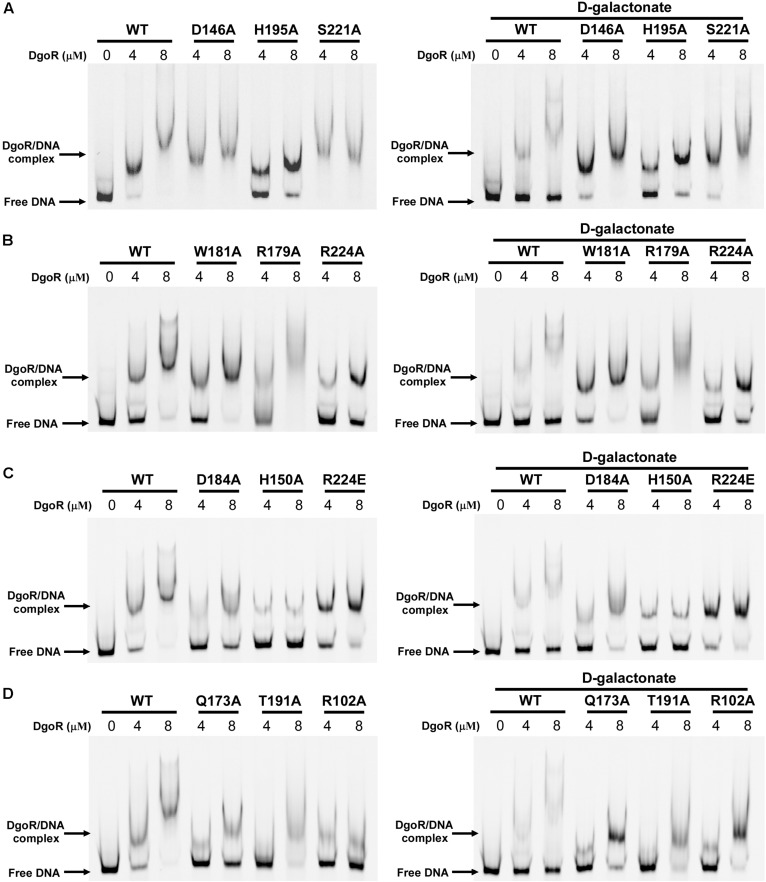
EMSA results of wild-type *Eco*DgoR and its mutants in the absence (left panel) and presence of 0.5 mM D-galactonate (right panel). **(A–D)**. The Cy5-labeled *dgo* cis-acting promoter region was incubated with the indicated concentrations of DgoR proteins (WT, R102A, D146A, H150A, Q173A, R179A, W181A, D184A, T191A, H195A, S221A, R224A, and R224E), and salmon sperm DNA was added in each sample to mask the nonspecific binding effect. The signals of free DNA and protein-DNA complexes were scanned and shown.

The EMSA assay was carried out using a 250 bp fragment (the *dgo* cis-acting element) from the promoter region of the *dgo* operon harboring *Eco*DgoR binding sites. It was observed that the binding of wild-type (WT) *Eco*DgoR protein to this region formed stable complex. The stability of the *Eco*DgoR-DNA complexes increased with the rise of *Eco*DgoR concentrations (up to 8 μM) in the binding reaction (Figure 5 and Supplementary Figure S1). After optimizing the binding conditions for WT *Eco*DgoR, the ability of *Eco*DgoR mutants to bind DNA was evaluated. The results obtained from EMSA (Figure 5) allowed the classification of mutants in three groups: Group I (R179A and W181A) shows no significant effect on the binding to DNA and D-galactonate; Group II (R102A, H150A, Q173A, D184A, T191A, H195A, R224A, and R224E) shows a decreased binding to both DNA and D-galactonate; Group III (D146A and S221A) shows increased DNA binding but can be slightly reverted in the presence of D-galactonate.

The Group I mutants R179A and W181A showed the similar DNA binding pattern to WT *Eco*DgoR without D-galactonate. Surprisingly, no significant difference in DNA binding affinity were observed either in the presence or absence of 0.5 mM D-galactonate, implying that the binding of D-galactonate may fail to allosterically regulate the DNA binding for these two mutants (Figure 5).

Compared to WT-DgoR, all the mutants in Group II exhibited decreased binding affinity to DNA at the concentrations of 4 and 8 μM as evidenced by the increased ratio of free DNA band and protein-DNA complex band, with the exception that R224E showed compromised DNA binding ability at 8 μM only. Particularly, the DNA binding ability of the mutants Q173A, R102A, D184A, T191A, and H150A was almost abolished as demonstrated by the faint bands for protein-DNA complex (Figure 5). Upon the addition of D-galactonate (0.5 mM), Q173A, R102A, D184A, and T191A restored their DNA binding ability to some extent at the concentration of 8 μM, while H150A, H195A, R224A, and R224E remain inability to bind DNA (Figure 5). These results indicated that the amino acid residues R102, H150, Q173, D184, T191, H195, and R224 may involve in the binding to D-galactonate.

The Group III mutants D146A and S221A displayed markedly increased DNA binding affinity at the concentration of 4 μM compared to WT-DgoR as illustrated by the missing band for free DNA (Figure 5). Supplementation of D-galactonate slightly decreased their ability to bind DNA, suggesting the Group III mutants D146A and S221A may not participate in the direct binding of D-galactonate.

**FIGURE 6:**
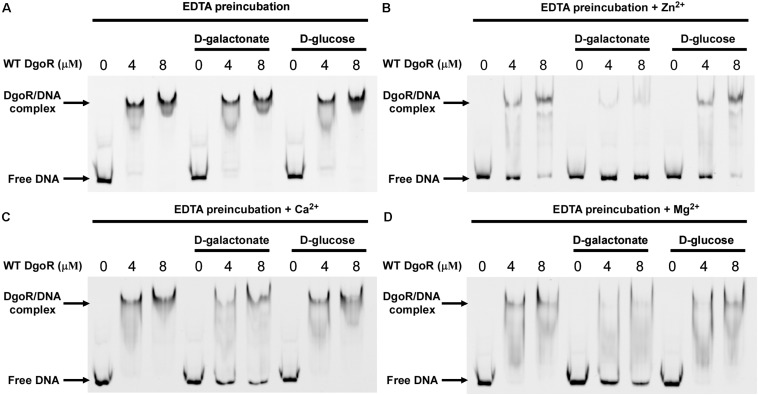
Effects of divalent metals on *Eco*DgoR binding to *dgo* cis-acting promoter DNA in the presence or absence of 0.5 mM D-galactonate or D-glucose. **(A–D)** WT *Eco*DgoR was pre-incubated with EDTA for 2 h at 4°C and then dialyzed overnight before EMSAs. Divalent metal ions are as indicated on top of each panel. Others are similar as panel B.

The three-blade propeller scaffold comprising of H150, H195 and D146 residues in the C-terminal domain of *Eco*DgoR is usually associated with divalent metal cation binding with high affinity. The mutation of D146, H150, or H195 to Ala resulted in a remarkably decreased binding of *Eco*DgoR to D-galactonate (Figure 5), indicating the three amino acid residues are critical to the coordination of divalent cations that further mediate ligand-protein interaction as proposed in our molecular docking study. To further examine the role of divalent metal cations in D-galactonate binding, *Eco*DgoR was incubated in the presence of 5 mM EDTA and subsequently subjected to EMSA analysis. Interestingly, the depletion of metal cations appeared to have no significant effect on the binding of *Eco*DgoR to the *dgo* cis-acting element in the absence or presence of D-galactonate or D-glucose; the addition of D-galactonate or D-glucose alone didn’t disrupt the formation of stable protein/DNA complexes (Figure 6A), while the supplementation with D-galactonate and selected divalent metal cations (i.e., Zn^2+^, Ca^2+^, or Mg^2+^) decreased the stability of *Eco*DgoR/DNA complex (Figures 6B–D). Conversely, the stability of *Eco*DgoR/DNA complexes was not affected significantly when D-glucose and each divalent metal cation were supplemented simultaneously, especially for Ca^2+^ and Mg^2+^ (Figures 6B–D). These results suggest that D-galactonate binding to *Eco*DgoR is mediated by a divalent metal cofactor, and the interactions are required for *Eco*DgoR binding to the *dgo* cis-acting element.

### Mutations in the C-Terminal of *Eco*DgoR Affect Its Thermal Stability

**FIGURE 7:**
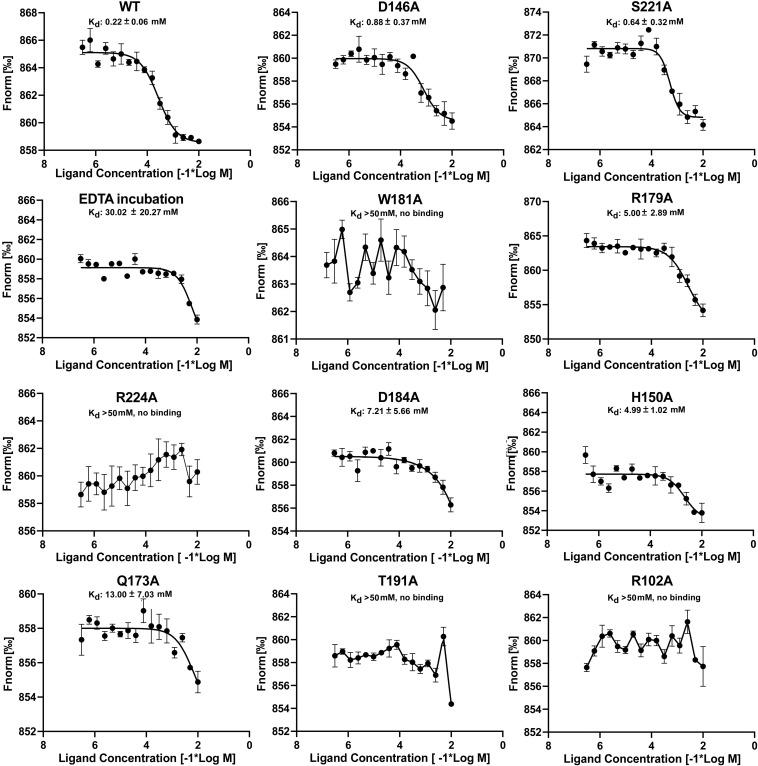
MST analyses of the binding of D-galactonate to wild-type and mutated *Eco*DgoR proteins with or without EDTA pre-incubation. The concentration of wild-type *Eco*DgoR and its indicated mutants is kept constantly at 100 nM, while the D-galactonate concentration varies from 305 nM to 10 mM. The binding disappeared for *Eco*DgoR mutants W181A, R224A, T191A, and R102A suggested by the almost flat curves.

EMSA assays indicated that specific amino acid substitutions affected the ability of *Eco*DgoR to bind DNA, as well as the binding of the effector molecule D-galactonate, Moreover, EDTA preincubation had significant effect on the binding of D-galactonate to *Eco*DgoR (Figures 5, 6). To identify the residues exclusively involved in D-galactonate binding, confirm the effect of divalent metal cation on the ligand-protein interaction, and further characterize the interactions of WT/mutated *Eco*DgoR with the effector molecule, the thermodynamic properties of interactions were determined using MST assays. The titration of *Eco*DgoR with D-galactonate followed an endothermal heat change profile, giving rise to a sigmoidal binding curve (Figure 7). The estimated dissociation constants K_*d*_ of WT-*Eco*DgoR and its mutants with D-galactonate using nonlinear fitting assuming one specific binding site were summarized in Figure 7. The K_*d*_ value of WT-*Eco*DgoR binding D-galactonate is at low millimolar range (∼0.22 mM), indicating a moderate affinity for the ligand. The decreased binding affinity of mutated proteins to D-galactonate was confirmed using MST assays. In agreement with the EMSA results, R102A, W181A, T191A, and R224A mutants did not interact with D-galactonate, while D146A, H150A, Q173A, R179A, D184A, and S221A had a decreased binding affinity to D-galactonate compared to WT-*Eco*DgoR. Both EMSA and MST results are fully consistent with the interactions revealed by the docking models, and suggest that key interactions from the proposed effector binding pockets are critical for divalent metal ion and D-galactonate binding.

## Discussion

*Eco*DgoR is classified as a member of FadR family in the GntR superfamily. In FadR family, the FCD domain highly likely binds divalent metal ion, which is important to stabilize the ligand located at the binding pocket. The effector binding domain (FCD domain) of *Eco*DgoR is composed of seven alpha-helices and is bound to metal ion, which resembles the binding pocket of *cgl*LldR protein (CGL2915). The polar ligand-binding cavity of *Eco*DgoR is significantly larger than other metal-ion binding FCD domains identified to date, with an estimated volume of 1,532 Å^3^. Such a binding pocket allows large effectors, such as D-galactonate, to enter and trigger the allosteric regulation. Previous study showed that the helix α4 appears to be a key component in conformation transmission (Gao et al., 2008; Zheng et al., 2009). Substrates binding to the cavity in the C-terminal domain triggers conformational changes of helix α8 surrounding the cavity, which causes a shift of helix α4 toward helix α1 in the N-terminal domain. This shift generates a rearrangement of the DNA binding domain, and the protein scaffold undergoes a dramatic conformational change. As a result, the protein decreases its affinity to DNA and consequently depresses transcription.

In the *Eco*DgoR C-terminal FCD domain, the cavity is surrounded by residues R102, Q173, R179, S221, W181, R224, D184, and T191, which may interact with D-galactonate directly suggested from molecular docking study. *Eco*DgoR residues R102, Q173, R224, T191, H150, and H195 are found to be essential for both DNA and ligand binding as determined by MST and EMSA; residues S221, W181, and R179 are important for ligand binding but have less impact on DNA binding.

The co-crystalized structures of several FadR family transcriptional factors bound with divalent metal ions have been determined (van Aalten et al., 2000, 2001a; Gao et al., 2008; Zheng et al., 2009; Adams et al., 2010; Fillenberg et al., 2016). All of them have a divalent metal ion coordinated by three conserved histidine residues (e.g., H134, H174, and H196 in *Tm*0439; H148, H192, and H214 in *Ps*5454) at the core of the helical bundle. Our study revealed that *Eco*DgoR D146, H150, and H195 residues, which are different from metal coordinating residues in all identified FCD domains, are involved in the divalent metal ion binding, the replacement of D146, H150 and H195 with alanine or EDTA treatment of *Eco*DgoR in the presence of D-galactonate did not display remarkable effects on DNA binding in EMSA assay, suggesting divalent metal ions do not regulate the interaction between *Eco*DgoR and DNA directly (Figures 5, 6). However, divalent metal ions are essential for D-galactonate to bind *Eco*DgoR as suggested by MST assay (Figure 7). These results suggest that divalent metal ions play an important role in D-galactonate-*Eco*DgoR interaction, which further trigger the allosteric regulation.

In conclusion, the structural and biochemical evidence presented here reveals the D-galactonate binding pocket in the FCD domain of *Eco*DgoR, and identifies the key amino acid residues that interact with divalent metal ions essential for D-galactonate-*Eco*DgoR binding. However, the divalent metal ions are not directly involved in the interaction between *Eco*DgoR and DNA. The residues in the large D-galactonate binding pocket of *Eco*DgoR are essential for ligand selectivity and stability. Compared to other FadR family transcription factors with a divalent metal ion, *Eco*DgoR provides not only a larger cavity that allows effector like D-galactonate to bind, but also a metal center with distinct components for its function. Our results here reveal a distinct example that could help the understanding of the structure and mechanism of metal ion-containing transcription factors.

## Data Availability Statement

The structure of EcoDgoR_C has been deposited into Protein Data Bank (PDB) with the accession number of 7C7E.

## Author Contributions

ZS, YC, ST, and WL designed experiments, analyzed the data, and wrote the manuscript. ZL, YS, and YL performed the bulk of the experiments, contributed to the protein expression, purification, and crystallization, and contributed to the EMSA experiments. YC contributed to molecular docking assay. WL conceived the project. All authors contributed to the article and approved the submitted version.

## Conflict of Interest

The authors declare that the research was conducted in the absence of any commercial or financial relationships that could be construed as a potential conflict of interest.
