# Supplementary material for: Structural and Functional Analyses of the Transcription Repressor DgoR From Escherichia coli Reveal a Divalent Metal-Containing D-Galactonate Binding Pocket
Source: Front Microbiol. 2020 Nov 5;11:590330. doi: 10.3389/fmicb.2020.590330 (PMC7674646; doi:10.3389/fmicb.2020.590330)
Supplement: Supplementary file 1 [file Data_Sheet_1.docx]

Supplementary Material

Structural and functional analyses of the transcription repressor DgoR from *Escherichia coli* reveal a divalent metal-containing d-galactonate binding pocket

**Zhaozhu Lin^1,#^, Yi Sun^1,#^, Yu Liu^5,#^, Shujuan Tong^1,*^, Zhuo Shang^1,*^, Yuanheng Cai^4, *^, Wei Lin^1,2, 3,*^**

^1^ Department of Microbiology and Immunology, School of Medicine & Holistic Integrative Medicine, Nanjing University of Chinese Medicine, Nanjing, China.

^2^ State Key Laboratory of Natural Medicines, China Pharmaceutical University, Nanjing, China.

^3^ Jiangsu Collaborative Innovation Center of Chinese Medicinal Resources Industrialization, Nanjing 210023, China.

^4^ Biochemistry and Cell Biology Department, Stony Brook University, Stony Brook, New York 11794, United States.

^5^ Waksman Institute of Microbiology and Department of Chemistry, Rutgers University, Piscataway, NJ, 08854, USA.

#, Equal contribution.

*** Correspondence:**Corresponding Author
[weilin@njucm.edu.cn](mailto:weilin@njucm.edu.cn), [caiy@bnl.gov](mailto:caiy@bnl.gov), [alexsz1985@gmail.com](mailto:alexsz1985@gmail.com) and [tongshujuan6@sina.com](mailto:tongshujuan6@sina.com)

**Supplementary Table S1 Bacterial strains, plasmids, and primers used in this study**

| Bacterial strains, plasmids, primers | Description | Source/Reference |
| --- | --- | --- |
| **Strains** |  |  |
| *E. coli* |  |  |
| DH5a | F-，φ80dlacZΔM15，Δ(lacZYA-argF)U169，deoR，recA1，endA1，hsdR17(rk-，mk+)，phoA，supE44，λ-，thi-1，gyrA96，relA1 | Invitrogen |
| BL21(DE3) | F-，ompT， hsdS（rBB-mB－），gal， dcm（DE3） | Invitrogen |
| **Plasmids** |  |  |
| pET32a | 5422bp, T7/lac, pBR322 ori, Kan, N-terminal His Tag/thrombin/ S Tag/enterokinase configuration plus an optional C-terminal His Tag | EMD Biosciences(Novagen) |
| **Primers** |  |  |
| DgoR_F | AAAGGATCCACTCTCAATAAAACCGATCGCATTGTC | This study |
| DgoRS_F | AAAGCTAGCAATTACCTCGACACTGACGTACTG | This study |
| DgoR_R | AAAAGCTTTCATGTGATTTCCTTTAACCTTCGTGT | This study |
| Cy5-M13F | TGTAAAACGACGGCCAGT | This study |
| *dgoR*cis_F | TGTAAAACGACGGCCAGTGCATTGTTCTTTTTGTGATCTAAATTGTAGTAC | This study |
| *dgoR*cis_R | GACAATGCGATCGGTTTTATTGAGAGT | This study |
| DgoRR102A_F | GCCATGAGCGAAGTGGCAAATCTGGTGGAACCG | This study |
| DgoRR102A_R | CGGTTCCACCAGATTTGCCACTTCGCTCATGGC | This study |
| DgoRD146A_F | GCGTTTAACGAAGCGGCTATTCGCTACCACGAG | This study |
| DgoRD146A_R | CTCGTGGTAGCGAATAGCCGCTTCGTTAAACGC | This study |
| DgoRD150A_F | GCGGATATTCGCTACGCCGAGGCGGTGCTGCAG | This study |
| DgoRD150A_R | CTGCAGCACCGCCTCGGCGTAGCGAATATCCGC | This study |
| DgoRQ173A_F | GCGATCAGTTCGCTGGAGCGGGCGGTTTTTGAA | This study |
| DgoRQ173A_R | TTCAAAAACCGCCCGCTCCAGCGAACTGATCGC | This study |
| DgoRR179A_F | CGGGCGGTTTTTGAAGCAACCTGGATGGGCGAT | This study |
| DgoRR179A_R | ATCGCCCATCCAGGTTGCTTCAAAAACCGCCCG | This study |
| DgoRW181A_F | GTTTTTGAACGAACCGCGATGGGCGATGAGGCC | This study |
| DgoRW181A_R | GGCCTCATCGCCCATCGCGGTTCGTTCAAAAAC | This study |
| DgoRD184A_F | CGAACCTGGATGGGCGCTGAGGCCAACATGCCG | This study |
| DgoRD184A_R | CGGCATGTTGGCCTCAGCGCCCATCCAGGTTCG | This study |
| DgoRT191A_F | GCCAACATGCCGCAAGCGCTCCAGGAACATAAG | This study |
| DgoRT191A_R | CTTATGTTCCTGGAGCGCTTGCGGCATGTTGGC | This study |
| DgoRH195A_F | CAAACGCTCCAGGAAGCTAAGGCGCTGTTCGAT | This study |
| DgoRH195A_R | ATCGAACAGCGCCTTAGCTTCCTGGAGCGTTTG | This study |
| DgoRS221A_F | ACCATGATCGCCAGCGCGACACGAAGGTTAAAG | This study |
| DgoRS221A_R | CTTTAACCTTCGTGTCGCGCTGGCGATCATGGT | This study |
| DgoRR224A_F | GCCAGCTCGACACGAGCGTTAAAGGAAATCACA | This study |
| DgoRR224A_R | TGTGATTTCCTTTAACGCTCGTGTCGAGCTGGC | This study |
| DgoRR224E_F | GCCAGCTCGACACGAGAGTTAAAGGAAATCACA | This study |
| DgoRR224E_R | TGTGATTTCCTTTAACTCTCGTGTCGAGCTGGC | This study |

**
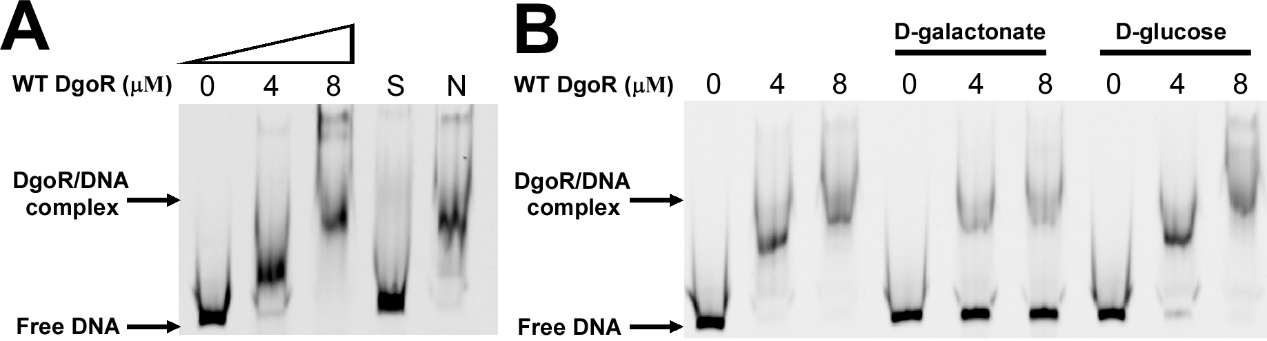
**

**Supplementary Figure S1. EMSAs of *Eco*DgoR binding to *dgo* cis-acting promoter DNA in the presence or absence of 0.5 mM d-galactonate or d-glucose. (A)** EMSAs of the wild-type (WT) *Eco*DgoR protein with *dgo* cis-acting promoter. EMSAs were carried out using the Cy5-labeled *dgo* cis-acting promoter region, which was incubated with the indicated concentrations of WT *Eco*DgoR proteins. Salmon sperm DNA was added into each sample to mask the non-specific binding effect. The signals of free DNA and protein-DNA complexes were scanned and shown. The specificity of bandshifts was verified by adding 100-fold excess of non-labelled specific probe DNA (S) and non-specific competitor DNA (sperm DNA) (N). The protein concentrations are used as follows: 0, 4, 8 μM. **(B)** EMSAs of WT *Eco*DgoR protein with *dgo* cis-acting promoter were performed and indicated concentrations of WT *Eco*DgoR protein (0, 4, 8 μM) in the presence or absence of d-galactonate or d-glucose (0.5 mM).


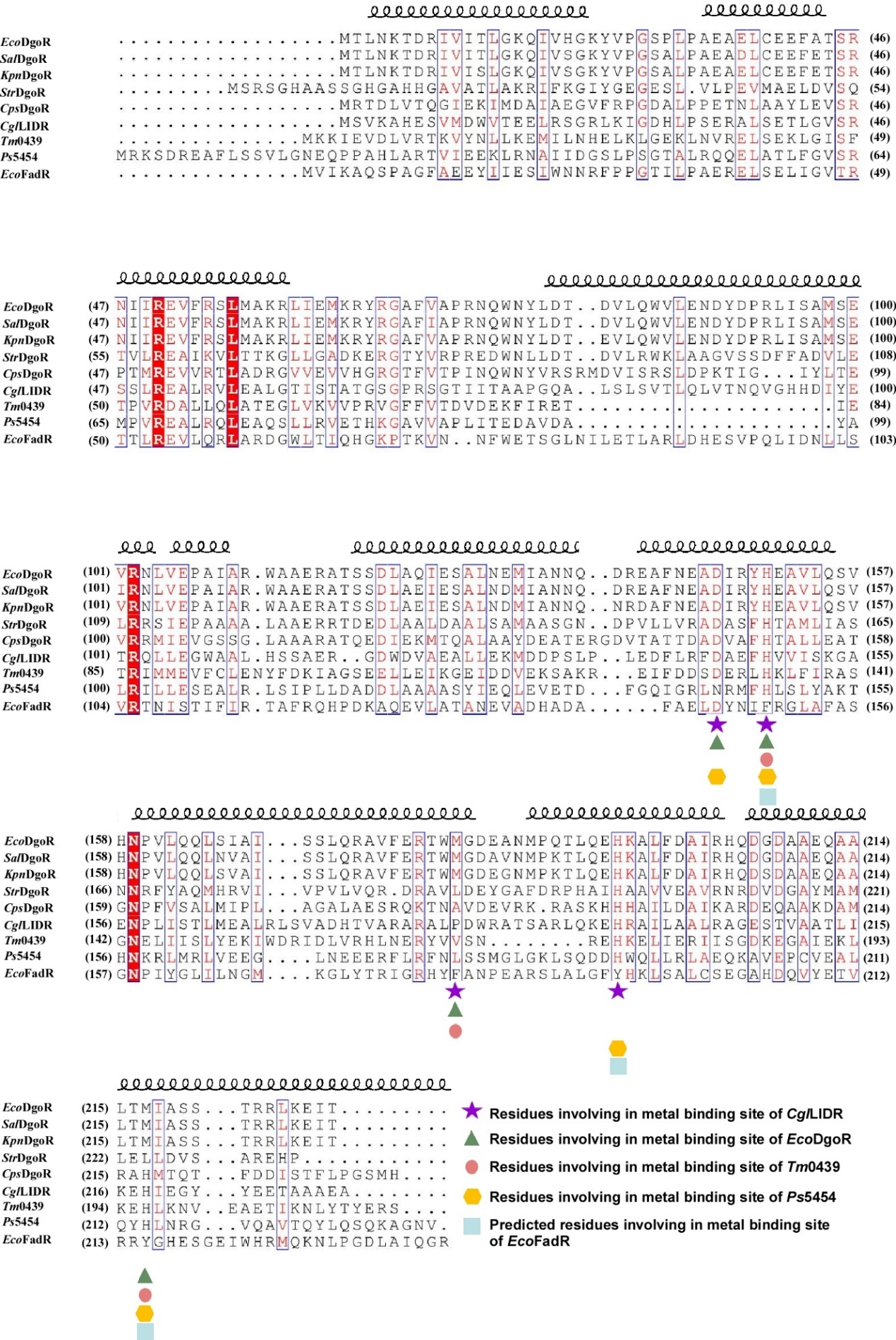


**Supplementary Figure S2.** **Structure-based sequence alignment of DgoR homologues, *Cgl*LIDR, *Tm*0439, *Ps*5454 and *Eco*FadR.** The invariant residues are highlighted in red, and conserved amino acids are boxed. residues in the metal binding site of *Eco*DgoR are indicated with purple stars; residues in the metal binding site of *Cgl*LIDR are indicated with green triangle; residues in the metal binding site of *Tm*0439 are indicated with pink cycle; residues in the metal binding site of *Ps*5454 are indicated with yellow regular hexagon; residues in the metal binding site of *Eco*FadR are indicated with blue square. The secondary structure elements of *Eco*FadR are shown at the top. *Eco*DgoR, DgoR from *Escherichia coli* strain K12; *Sal*DgoR, DgoR from *Salmonella enterica* *subsp. enterica serovar Typhi* strain CT18; *Kpn*DgoR, DgoR from *Klebsiella pneumonia subsp. Rhinoscleromatis* strain SB3432; *Str*DgoR, DgoR from *Streptomyces sp.* strain NBRC 110027; *Cps*DgoR, DgoR from *Corynebacterium pseudotuberculosis* strain FRC41; *Cgl*LIDR (PDB: 2DI3), GntR superfamily protein from *Corynebacterium glutamicum* strain ATCC 13032; *Tm*0439 (PDB: 3fms), GntR superfamily protein from *[Thermotoga maritima](https://www.rcsb.org/pdb/search/smartSubquery.do?smartSearchSubtype=TreeEntityQuery&t=1&n=243274)* [strain](https://www.rcsb.org/pdb/search/smartSubquery.do?smartSearchSubtype=TreeEntityQuery&t=1&n=243274)[ATCC 43589](https://www.rcsb.org/pdb/search/smartSubquery.do?smartSearchSubtype=TreeEntityQuery&t=1&n=243274); *Ps*5454 (PDB: 3c7J), GntR superfamily protein from *[Pseudomonas syringae](http://www.rcsb.org/search?q=rcsb_entity_source_organism.ncbi_scientific_name:Pseudomonas%20syringae%20pv.%20tomato%20str.%20DC3000)* [pv. tomato str. DC3000](http://www.rcsb.org/search?q=rcsb_entity_source_organism.ncbi_scientific_name:Pseudomonas%20syringae%20pv.%20tomato%20str.%20DC3000); *Eco*FadR, FadR from *Escherichia coli* strain K12.
